# The Brief Case: How antibiotic pretreatment complicated the diagnosis of *Haemophilus influenzae* type a meningitis

**DOI:** 10.1128/jcm.00540-25

**Published:** 2025-10-08

**Authors:** Kristen L. Buehne, Lauren Lajos, Bert Lopansri, Abby Tate, Christian L. Carlson, Diego Cerbian Chaustre, Sonia Mehra

**Affiliations:** 1 Department of Pediatrics, University of Utah Health 14434, Salt Lake City, Utah, USA; 2 Division of Pediatric Infectious Diseases, University of Utah Health 14434, Salt Lake City, Utah, USA; 3 Division of Infectious Diseases and Clinical Epidemiology, Intermountain Health 7061, Salt Lake City, Utah, USA; 4 Department of Medicine, University of Utah 7060, Salt Lake City, Utah, USA; 5 Department of Microbiology, Primary Children’s Hospital, Salt Lake City, Utah, USA; 6 Department of Pediatric Neuroradiology, Primary Children’s Hospital, Salt Lake City, Utah, USA; 7 Department of Neuroradiology, University of Utah Health 14434, Salt Lake City, Utah, USA; Mayo Clinic Minnesota, Rochester, Minnesota, USA

**Keywords:** meningitis, pretreatment, *Haemophilus influenzae*, spheroplast

## CASE

An 11-month-old unvaccinated male with Sotos syndrome, a genetic syndrome with learning disability, distinctive facial features, and head overgrowth, presented to the emergency department with poor oral intake, cough, congestion, and increased work of breathing. The diagnostic evaluation included complete blood count, comprehensive metabolic panel, C-reactive protein (CRP), erythrocyte sedimentation rate (ESR), chest X-ray, blood culture, and viral respiratory panel. Labs were significant for elevated inflammatory markers (CRP 27.7 [normal range 0–1 mg/dL], ESR 56 [normal range <20 mm/h]). He was admitted for IV hydration for presumed viral illness.

**Fig 1:**
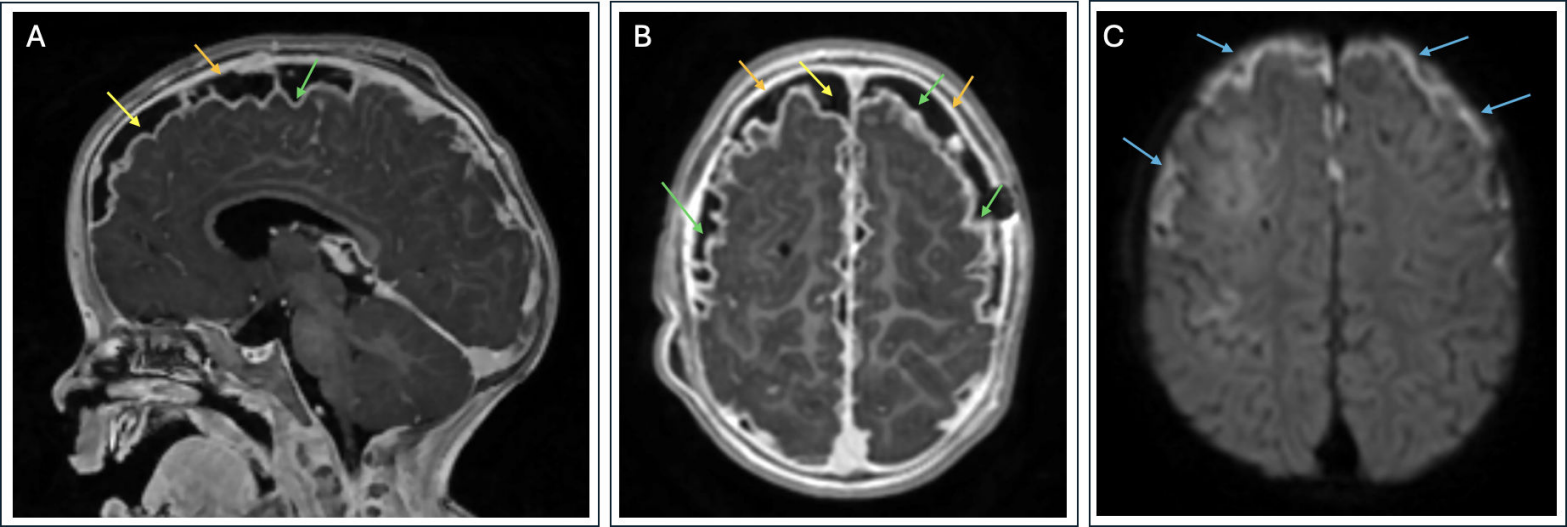
Brain MRI with and without contrast. Image (**A**) displays a sagittal T1-weighted post-contrast image; image (**B**) is an axial T1-weighted post-contrast image; and image (**C**) is an axial diffusion-weighted image. There is diffuse leptomeningeal (green arrows) and pachymeningeal (orange arrows) enhancement associated with bilateral hypoenhancing fluid collections along the cerebral convexities (yellow arrows) displaying restricted diffusion (blue arrows). This is consistent with meningitis complicated by subarachnoid empyema.

By the next day, the patient became increasingly lethargic with worsening inflammatory markers (CRP 41.3 mg/dL), leukopenia (WBC 2.0 K/mcL), and neutropenia (ANC 0.8 K/mcL). The CT head was unrevealing, but with concern for CNS infection, he was empirically started on IV vancomycin, IV acyclovir, and IV ceftriaxone at meningitic dosing. The initial lumbar puncture (LP) done 6 h after the first dose of antibiotics was remarkable for glucose <1 mg/dL (normal range 40–70 mg/dL), protein 219 mg/dL (normal range 12–60 mg/dL), and WBC 243 K/mcL (24% bands, 45% neutrophils). Gram stain from the cerebrospinal fluid (CSF) showed Gram-negative rods. Both blood and CSF cultures grew *Haemophilus influenzae* within approximately 24–36 h of collection, with gray, raised, smooth, mucoid-appearing colonies on a chocolate agar plate. The organism was identified via MALDI-TOF mass spectrometry, and beta-lactamase testing was positive. Further antibiotic susceptibility testing was not performed, and the isolate was sent to the state health department for typing. Brain MRI was consistent with diffuse leptomeningeal enhancement with bilateral subarachnoid empyema, right greater than left (Fig. 1).

**Fig 2:**
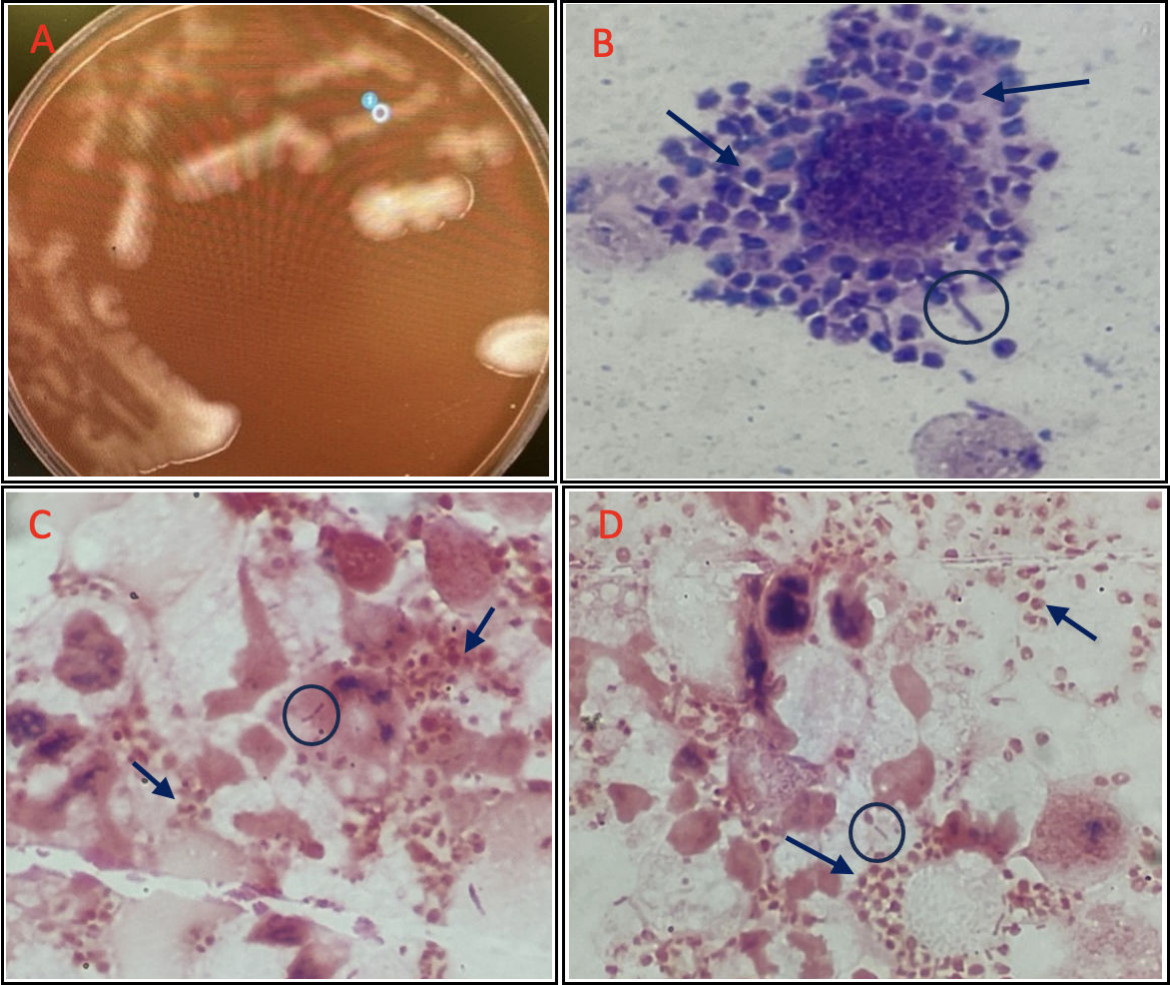
(**A**) Growth of *H. influenzae* on a chocolate agar plate around 80 h old from our patient’s CSF culture from his initial LP. The blue dot and white circle represent the colonies selected for additional workup on the automated line. (**B**) A Wright stain performed on our patient’s CSF specimen during the initial pathology review demonstrating the extracellular, round structures postulated to be spheroplasts (arrows), along with elongated, Gram-negative organisms (encircled). (**C and D**) Gram stain images from our patient’s CSF specimen obtained via his initial LP. Elongated structures thought to be the Gram-negative bacteria are encircled. Arrows indicate the retrospectively appreciated intracellular and extracellular round structures that are hypothesized to be *H. influenzae* type a spheroplasts pretreated with ceftriaxone.

Despite findings consistent with *H. influenzae* meningitis and bacteremia, diagnostic uncertainty arose after large, unidentifiable structures were identified during CSF cytology analysis, prompting further pathological review (Fig. 2). In addition to Gram-negative rods, pathology review identified numerous Gram-negative, intracellular bacteria and extracellular structures of uncertain significance, with the morphology resembling yeast. This raised concern for possible laboratory contamination, fungal or parasitic co-infection, and/or underlying primary immunodeficiency despite the patient’s lack of exposures or recurrent past infections. Opportunity for sample and/or reagent contamination was eliminated in discussion with lab personnel. Wright and Giemsa staining also captured the structures in question and were suggestive of a possible capsule around the organism. Calcofluor stains were negative. Extensive testing for the fungal, parasitic, and immunodeficiency concerns was negative. Consequently, with these findings and expert consultation with infectious disease and microbiology colleagues, the unusual structures identified were postulated to most likely resemble “spheroplasts” (i.e., morphologically altered *H. influenzae* secondary to antibiotic exposure).

With organism identification consistent with *H. influenzae* and positive beta-lactamase testing, antimicrobials were narrowed to IV ceftriaxone. The health department identified his isolate as *H. influenzae* type a. His course was complicated by subarachnoid empyema requiring emergent EVD placement as well as drainage of empyemas, suspected watershed infarcts, EEG-negative focal seizures/myoclonus, and bilateral hearing loss requiring cochlear implants and 4 days of dexamethasone. Ultimately, he completed a total of 8 weeks of antibiotic therapy. Aside from hearing loss, he returned to his neurologic baseline 6–7 months later.

## DISCUSSION

*H. influenzae* is a facultatively anaerobic Gram-negative coccobacillus, a common commensal in the human nasopharynx (1). These bacteria grow well on chocolate agar largely due to the needed release of X factor (hematin) and V factor (phosphopyridine nucleotide) secondary to the lysis of red blood cells during the preparation of chocolate agar. Growth can also be elicited on the BVCCA medium, which contains antibiotics (bacitracin, vancomycin, and clindamycin) in addition to chocolate agar constituents.

*H. influenzae* strains consist of those that are typeable, distinguished by the presence of a polysaccharide capsule, and non-typeable. There are six unique capsules (types a–f) that correlate with the six typeable *H. influenzae* serotypes. Our patient’s *H. influenzae* organism was ultimately serotyped as type a by the health department. All of the serotypes, particularly type b (Hib), are common etiological agents in lower respiratory tract infections, but they can also cause other serious infections, such as meningitis, epiglottitis, cellulitis, septic arthritis, osteomyelitis, and bacteremia (1). Since the implementation of the conjugate Hib vaccine in the late 1980s, rates of invasive Hib disease have markedly plummeted. Resultantly, non-typeable *H. influenzae* (NTHi), a common cause of acute otitis media and sinusitis, is currently the leading cause of invasive *H. influenzae* disease (2).

*H. influenzae* type a (Hia), while overall rare, is on the rise in both European countries and across northern North America, most notably among Indigenous American and Alaska Native populations (3). Of the typeable *H. influenzae*, Hia is now the most common encapsulated serotype causing invasive disease in children younger than 5 years (2). Explanations for this phenomenon remain vague, with hypotheses of overcrowding, poverty, and air quality (3). Clinical manifestations of invasive Hia disease are similar to Hib, typically causing meningitis and bacteremia in young children (4, 5). These characteristics of invasive Hia disease, along with high case-fatality rates, are reminiscent of invasive Hib disease in the pre-vaccine era (6). Encouragingly, Hia vaccination development is in progress with a phase 1 trial underway in Canada (7).

Diagnosing invasive *H. influenzae* disease is typically achieved via culture growth on appropriate media from CSF, blood, synovial fluid, pleural fluid, or pericardial fluid (2). Obtaining CSF in disseminated disease should be strongly considered, even without clinical suspicion for CNS involvement (2). Agglutination with antiserum or capsular typing with polymerase chain reaction (PCR) is often done for serotyping. In children diagnosed with invasive NTHi disease, an immunodeficiency workup could be considered to investigate the etiology of severe infection (1). Of note, our patient presented with a pre-diagnosed genetic syndrome, Sotos syndrome; this condition has not been associated with known immunodeficiencies to date, and our patient’s immune workup was unrevealing (8, 9).

Interestingly, the presence of “spheroplasts” on staining in the setting of antibiotic pretreatment, as likely observed in this case, has been previously described. Specifically, the Bottone group in 1976 used the term “hazy” to describe the *H. influenzae* growth observed in culture tubes treated with antibiotics. This appearance was noted only when the bacteria were at high concentrations and mixed with ampicillin, penicillin, or cephalothin (10). On microscopic examination of this “hazy” culture fluid, the team examined “spheroplasts” or large spherical bodies of serotypeable *H. influenzae*, rather than the classic coccobacillus. Notably, cultures without exposure to beta-lactams revealed the classic morphology for *H. influenzae. Escherichia coli*, another encapsulated Gram-negative organism, has more recently also been noted to morph while undergoing treatment with a beta-lactam antibiotic, specifically via a four-stage process: elongation, bulge formation, bulge stagnation, and lysis (11). We speculate that the unusual morphology noted in this patient’s case, which caused diagnostic uncertainty, was likely, although not definitively, due to the bacteria’s exposure to a beta-lactam antibiotic.

Regarding treatment, a third-generation cephalosporin remains the foundation for *H. influenzae* invasive disease while awaiting culture and susceptibility results. For Hib meningitis, dexamethasone is an important adjunctive treatment if administered before or concurrently with the first dose of antimicrobial agent to reduce the risk of hearing loss (1, 2). Due to the limited number of available clinical pediatric studies, the utility of steroids beyond the period of initial antibiotic administration is not well understood. The benefit of steroids with other strains of *H. influenzae* is also not well described. Chemoprophylaxis with rifampin may also be indicated in specific situations, particularly those involving household contacts under 4 years of age, the immunocompromised, and preschools (2).

As trends in vaccine hesitancy increase, and Hia rates continue to rise, it is important for microbiologists and infectious disease providers to understand that a spheroplastic appearance on a pretreated, encapsulated specimen could potentially occur. Without a general appreciation for this morphological phenomenon, medical providers in similar clinical situations may struggle with diagnostic uncertainty, prompting excessive investigation and overly broad treatments in otherwise classic disease presentations. This case provides supporting evidence to a growing body of clinically relevant microbiological knowledge regarding infections with encapsulated Gram-negative bacteria under antimicrobial stress, and facilitates the promotion of judicious diagnostic and therapeutic stewardship practices in the future.

## SELF-ASSESSMENT QUESTIONS

1. What two factors are needed to grow *H. influenzae* on culture?
  - hematin, phosphopyridine nucleotide
  - hexokinase, lactose
  - phosphopyridine nucleotide, methylene blue
  - lactose, hematin
2. At __ bacterial concentrations and ___ beta-lactam exposure, atypical *H. influenzae* morphology, including a “hazy” appearance or “spheroplast” formation, can be appreciated.
  - high, without
  - low, without
  - high, with
  - low, with
3. Empiric treatment for *H. influenzae* type b meningitis includes:
  - ampicillin and dexamethasone simultaneously
  - third-generation cephalosporin
  - fluoroquinolone plus dexamethasone 24 h after the first antibiotic
  - third-generation cephalosporin plus dexamethasone prior to antibiotic administration

## ANSWERS TO SELF-ASSESSMENT QUESTIONS

1. What two factors are needed to grow *H. influenzae* on culture?
  - hematin, phosphopyridine nucleotide
  - hexokinase, lactose
  - phosphopyridine nucleotide, methylene blue
  - lactose, hematin

Answer: a. X factor (hematin) and V factor (phosphopyridine nucleotide) are needed to grow *H. influenza*. Hexokinase, lactose, and methylene blue are not needed.

- At __ bacterial concentrations and ___ beta-lactam exposure, atypical *H. influenzae* morphology, including a “hazy” appearance or “spheroplast” formation, can be appreciated.
  - high, without
  - low, without
  - high, with
  - low, with

Answer: c. At high bacterial concentrations and with beta-lactam exposure, Bottone’s group observed these findings in *H. influenzae* (10).

- Empiric treatment for *H. influenzae* type b (Hib) meningitis includes:
  - ampicillin and dexamethasone simultaneously
  - third-generation cephalosporin
  - fluoroquinolone plus dexamethasone 24 h after the first antibiotic
  - third-generation cephalosporin plus dexamethasone prior to antibiotic administration

Answer: d. A third-generation cephalosporin in combination with dexamethasone, administered either prior to or simultaneously with antibiotics, is the empiric treatment for Hib. Dexamethasone administered before or concurrently with empiric antibiotics is intended to minimize the risks of sensorineural hearing loss. Ampicillin could be used pending bacterial susceptibilities and other patient factors, but it is not an appropriate empirical therapy due to the potential beta-lactamase-producing abilities of *H. influenzae*. Fluoroquinolones are not preferred for empiric treatment in part due to their side effect profile.

**TAKE HOME POINTS**

- *H. influenzae* type a infections are on the rise and can present similarly to Hib infections in terms of severe illness, such as meningitis or bacteremia.
- Specimens of *H. influenzae* that are pretreated with a beta-lactam antibiotic may induce a “spheroplastic” (rather than the classic coccobacilli) appearance on Gram stain.
- Empiric treatment for *H. influenzae* infections includes a third-generation cephalosporin. For Hib meningitis, adding dexamethasone prior to or concurrently with the first dose of antibiotic treatment can be an essential adjunct to preventing hearing loss.
